# Myasthenic Crisis in a Patient Initially Treated for Transient Ischaemic Attack and Infection: A Case Report

**DOI:** 10.7759/cureus.93511

**Published:** 2025-09-29

**Authors:** Aditya K Adhikarla

**Affiliations:** 1 Internal Medicine, Manchester Royal Infirmary, Manchester, GBR

**Keywords:** atypical presentation, intravenous immunoglobulins (ivig), myasthenia gravis crisis, myasthenia gravis (mg), ptosis

## Abstract

Myasthenia gravis (MG) is the most common disorder of the neuromuscular junction and may present with fluctuating ocular, bulbar, or limb weakness. Myasthenic crisis represents a life-threatening complication characterised by respiratory failure requiring ventilatory support. We report the case of a 46-year-old South Asian man who initially presented with fluctuating bilateral ptosis, intermittent diplopia, and fatigue, and was managed as a transient ischaemic attack and respiratory tract infection. He subsequently deteriorated with hypoxia, extensive secretion burden, and pneumonia, necessitating intubation and intensive care admission. Despite broad-spectrum antibiotics and supportive care, ventilatory requirements persisted. Following neurological review and a more detailed history, together with multidisciplinary discussion, a myasthenic crisis was suspected, and treatment with intravenous immunoglobulin and corticosteroids was initiated. Acetylcholine receptor antibodies later returned positive, and electromyography demonstrated a decremental response, confirming the diagnosis. The patient improved gradually, was extubated on Day 14, and discharged with immunosuppressive therapy and neurology follow-up. This case underscores the importance of thorough neurological examination and careful history-taking to support early diagnosis, alongside timely multidisciplinary team input to guide management.

## Introduction

Myasthenia gravis (MG) is an autoimmune neuromuscular disorder characterised by fluctuating weakness and fatigability of the ocular, bulbar, limb, or respiratory muscles [1]. It may occur at any age and has a worldwide distribution without geographic predilection, although onset in early childhood or after the age of 70 is relatively uncommon. The reported incidence ranges from 0.04 to 5 per 100,000 population per year. Demographic patterns differ between generalised and ocular subtypes: generalised MG (GMG) is more common in women, with a female-to-male ratio of at least 3:2, whereas ocular MG (OMG) more often affects men, particularly over the age of 40 [2]. The disease is usually driven by autoantibodies against acetylcholine receptors (AChRs) on the postsynaptic membrane of skeletal muscle, though less commonly antibodies against muscle-specific kinase (MuSK), low-density lipoprotein receptor-related protein 4 (LRP4), or agrin are implicated [3].

MG can be further classified into subgroups based on clinical features and antibody status, each carrying prognostic and therapeutic relevance. Early-onset MG occurs before the age of 50 and is associated with thymic hyperplasia, whereas late-onset MG presents after the age of 50 and is typically linked to thymic atrophy. Other subtypes include thymoma-associated MG, anti-MuSK antibody-positive MG, ocular MG limited to periocular muscle involvement, and seronegative MG, in which neither AChR nor MuSK antibodies are detected [4].

Myasthenic crisis is the most severe manifestation of the disease, presenting with rapidly progressive bulbar weakness, dysphagia, and respiratory muscle involvement that may progress to type 2 respiratory failure. This is a medical emergency requiring ventilatory support in an intensive care setting.

The management of MG rests on three key approaches. Acetylcholinesterase inhibitors such as pyridostigmine provide symptomatic benefit by enhancing neuromuscular transmission. Long-term immunosuppressive therapy with corticosteroids or agents such as azathioprine and mycophenolate mofetil is used to suppress the autoimmune process. In acute deterioration or crisis, rapid immunomodulatory therapies, including intravenous immunoglobulin (IVIG) or plasma exchange, are employed to neutralise or remove circulating antibodies. Thymectomy is recommended for all patients with MG associated with thymoma, with only rare exceptions [5].

We report a 46-year-old man with MG who initially presented with fluctuating ptosis and diplopia, managed as a transient ischaemic attack and infection, before further review confirmed the diagnosis of myasthenic crisis.

## Case presentation

A 46-year-old South Asian man presented to his general practitioner on the same morning that he developed bilateral ptosis and intermittent diplopia (direction not specified), along with a two-week history of fatigue. On initial examination, bilateral ptosis (asymmetry not specified) and diplopia were noted, while the remainder of the cranial nerve assessment was normal. He denied limb weakness, dysphagia, or speech disturbance. In view of his acute neurological symptoms and subtle behavioural changes, he was referred urgently to the emergency department (ED) for further evaluation to exclude a neurovascular cause.

His past medical history included type 2 diabetes mellitus, mild learning disability, schizophrenia, and depression. He was also an active smoker with a smoking history of more than 20 years.

In the ED of a tertiary care centre on the afternoon of the same day, he was reviewed and was found to be haemodynamically stable and afebrile, with oxygen saturation 97% on room air, blood pressure 138/80 mmHg, and respiratory rate 20/min. No features of diplopia, pupillary changes, or other neuro-ophthalmological signs were elicited on examination. Notably, the initial symptoms of ptosis and diplopia described to the GP were not documented during the ED assessment, though fatigability of the ocular muscles was not specifically assessed at that time. He was alert and orientated, with no limb weakness, sensory deficits, or gait abnormality. He reported ongoing fatigue and a mild cough for approximately two weeks; however, there were no clinical signs of infection on examination. His condition was attributed to a respiratory tract infection.

**Figure 1:**
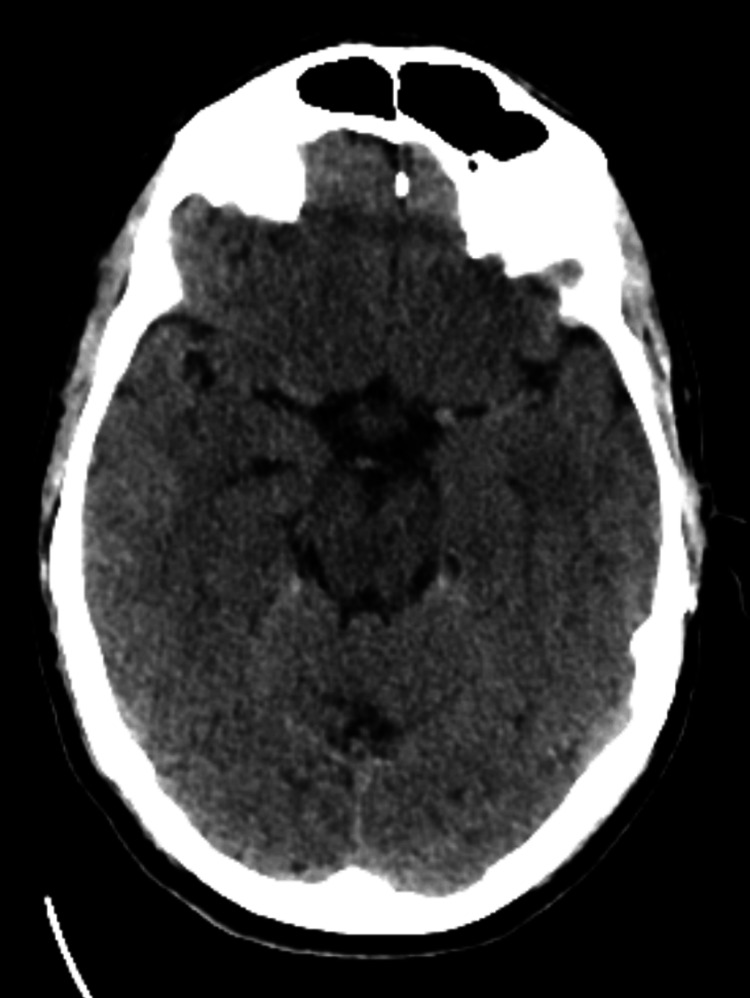
Axial non-contrast CT head demonstrating no acute intracranial abnormality. CT: computed tomography

As the bilateral ptosis and diplopia had resolved within a day of onset, and given his risk factors of diabetes and active smoking, in the absence of trauma or ophthalmological disease, a transient ischaemic attack (TIA) was suspected. A non-contrast brain CT showed no evidence of infarction or haemorrhage (Figure 1). He was reviewed by the stroke team, and an urgent outpatient brain MRI was requested. He was commenced on loading-dose aspirin 300 mg once daily. Routine blood tests were largely unremarkable, except for elevated inflammatory markers, with a C-reactive protein (CRP) level of 88 mg/L. Empirical treatment with oral co-amoxiclav 625 mg three times a day was initiated for a possible respiratory tract infection. Routine blood testing, including thyroid function tests, was normal, and both chest radiography and electrocardiogram were unremarkable.

With a working diagnosis of TIA and upper respiratory tract infection, the patient was discharged on a 14-day course of oral aspirin 300 mg once daily, empirical oral antibiotics, and urgent follow-up in the specialist stroke clinic for a brain MRI.

Four days later, he deteriorated at home with transient unresponsiveness and was brought to the hospital by ambulance. On arrival, he was hypoxic with oxygen saturations of 84% on room air, heart rate 110/min, respiratory rate 26/min, and a Glasgow Coma Scale (GCS) of 13. His blood pressure was steadily declining and was at 96/64 mmHg hg He also had an extensive secretion burden and was noted to be using accessory muscles of respiration. Clinical examination revealed bilateral basal crackles, bilateral ptosis, and slurred speech, but no meningism. He was promptly intubated, ventilated, and transferred to the intensive care unit (ICU). Arterial and central venous access were established, and a nasogastric tube was inserted on Day 1 of ICU admission. He was initially commenced on metaraminol via peripheral access, followed by noradrenaline support via central line to maintain haemodynamic stability in the context of sepsis.

**Figure 2:**
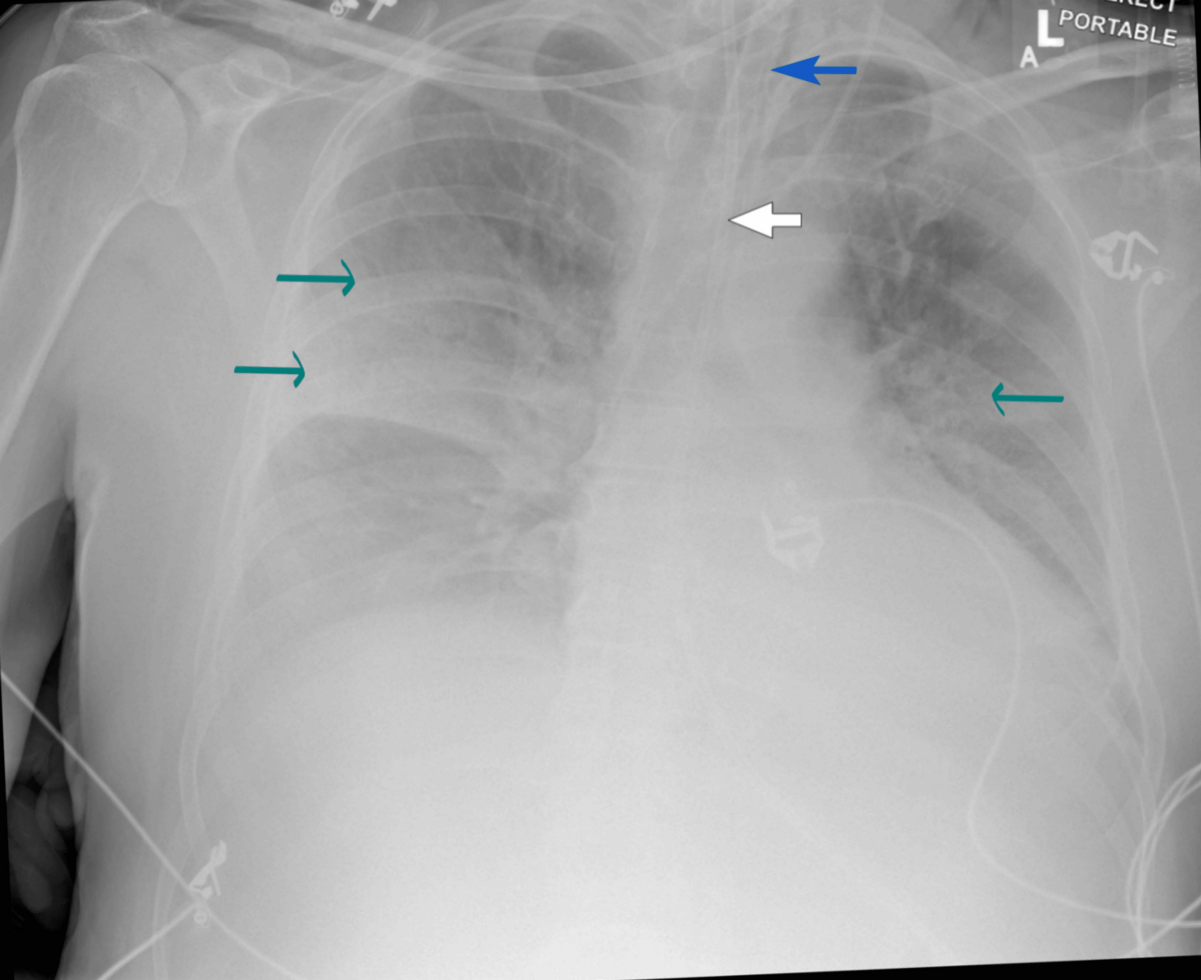
Chest X-ray (anteroposterior view) demonstrating bilateral pneumonic infiltrates Blue arrow: endotracheal tube; white arrow: nasogastric tube; green arrows: lobar infiltrates

Chest radiography demonstrated bilateral lobar consolidation (Figure 2). Inflammatory markers and white cell counts were elevated. Arterial blood gas confirmed type 1 respiratory failure in the context of pneumonia (Table 1). He was commenced on intravenous piperacillin-tazobactam (4.5 g three times daily) for presumed community-acquired pneumonia. A repeat brain CT performed on Day 1 in the ICU was again unremarkable. On Day 2, a lumbar puncture was undertaken to exclude meningoencephalitic causes of deterioration, including Guillain-Barré syndrome; results were unremarkable (Table 1). The aspirin was continued while requesting further input from the stroke team.

**Table 1:** Laboratory parameters on ICU admission

Test	Results	Reference range
Haemoglobin (hb)	14.4 g/dL	13.8-17.2 g/dL
White cell count (WCC)	17,000 cells/mcL	4,500-11,000 cells/ mcL
Neutrophils	8800 cels/mcL	2000-7,500 cells/mcL
Sodium (Na)	137 mmol/L	135-145 mmol/L
Potassium (K)	4.4 mmol/L	3.5-5.1 mmol/L
Urea	8.2 mmol/L	2.5–7.8 mmol/L
Creatinine	2.3	0.6-1.2mg/dl
Magnesium (Mg)	0.83 mmol/L	0.7-1.0 mmol/L
Adjusted calcium (Adj Ca)	2.40 mmol/L	2.2-2.6 mmol/L
Phosphate	0.94 mmol/L	0.8-1.5 mmol/L
C-reactive protein (CRP)	180 mg/L	<10 mg/L
Lactic acid	3.5 mmol/L	0.5-2.2 mmol/L
pH	7.36	7.35-7.45
pO2	7.8	10-13 kPa
Bicarbonate	23 mmol/L	22-28 mmol/L
Blood culture	Negative growth after 48 hours incubation	No growth
Creatine kinase	398 U/L	40-320 U/L
Cerebrospinal fluid (CSF) appearance	Clear, colourless	Clear, colourless
CSF white cell count	3 lymphocytes	<5 lymphocytes
CSF red cell count	2	0
CSF opening pressure	20	0-25 cmH₂O
CSF glucose	3.8 mmol/L	2.2-3.9 mmol/L
CSF lactate	2.3	1.2-2.1 mmol/L
CSF oligoclonal bands	Absent	Absent
CSF culture	No growth	No growth
Capillary blood glucose	7.6	4-7.8 mmol/L

An urgent neurology consultation was requested, given the complexity of symptoms and presentation. On review in the ICU, neurological examination demonstrated normal tone and reflexes, although assessment was limited as the patient was intubated and sedated. Collateral history from the patient’s next of kin revealed a three-week history of intermittent fatigue, more pronounced later in the day, along with low mood and fluctuating visual symptoms of diplopia preceding admission.

In line with neurology advice, further investigations were performed on Day 2, including autoimmune and vasculitis screens, acetylcholine receptor (AChR) antibodies, and anti-MuSK antibodies. Electromyography was difficult to perform and interpret at this stage due to intubation and sedation. Although inflammatory markers had improved (CRP 76 mg/L, WCC 11,200/µL) and inotropic support was being reduced, secretion burden and ventilatory requirements persisted. Following multidisciplinary discussion with the neurology team, a myasthenic crisis was suspected, and the patient was commenced on IVIG 0.4 g/kg/day on Day 5 of ICU admission for a five-day course. In addition, prednisolone 60 mg once a day was initiated via nasogastric tube. Screening blood tests were expedited, and AChR antibodies returned positive on Day 6 at 1 nmol/L (reference range 0-0.4 nmol/L), while the remainder of the autoimmune panel, including anti-MuSK antibodies, was negative (results available on Day 9).

In terms of ICU care, the patient underwent regular suctioning for oropharyngeal secretions. Ventilatory settings were gradually weaned, and he was extubated on Day 14 after a successful spontaneous breathing trial, once the secretion burden had improved.

**Figure 3:**
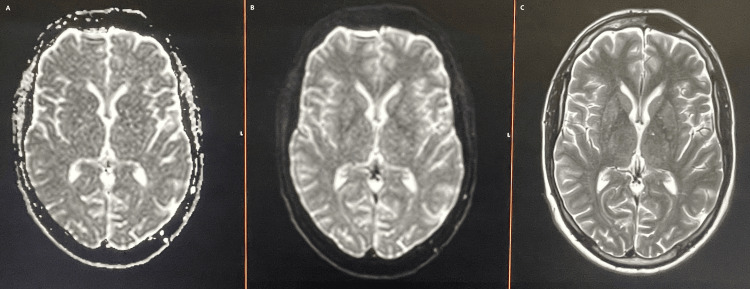
MRI head, axial sections: ADC (left), DWI (centre), T2 (right), showing no abnormality MRI: magnetic resonance imaging; ADC: apparent diffusion coefficient; DWI: diffusion weighted imaging

During his ICU stay, he was managed with a variable rate insulin infusion for glycaemic control under the diabetes nursing team and established on nasogastric feeding following dietetic input. He was also reviewed by the speech and language therapy (SALT) and stroke teams. Following extubation, a brain MRI performed on Day 15 demonstrated no focal abnormalities (Figure 3). His antiplatelet therapy was subsequently discontinued in view of the normal brain imaging and the confirmed alternative diagnosis, with subsequent clinical improvement.

The patient remained in the intensive care unit for 15 days and was transferred to the ward on Day 16, with regular monitoring of his vital signs. While on the ward, he underwent electromyography of the trapezius and deltoid muscles, which demonstrated a decremental response, further supporting the diagnosis of MG. With physiotherapy and supportive care, he made steady progress, with overall improvement in ocular, speech, and limb weakness, though some fatigable weakness persisted.

During his step-down to the ward, he was commenced on azathioprine 25 mg once daily (increased to 50 mg after one week), following confirmation of normal thiopurine methyltransferase (TPMT) activity on Day 16, after a delay in obtaining the results. He was initiated on pyridostigmine 60 mg four times daily, while oral prednisolone was continued with concurrent gastroprotective and bone-protective measures.

He was reviewed by the diabetes specialist team, and his regimen was optimised with the initiation of gliclazide 40 mg once daily and an increase in metformin to 1 g twice daily with a plan for outpatient follow-up. He was discharged after 20 days in hospital, with outpatient neurology follow-up arranged in 4-6 weeks. Outpatient investigations included a CT thorax, which excluded thymoma.

## Discussion

MG is the most common disorder of the neuromuscular junction and may present with myasthenic crisis and acute respiratory failure requiring intensive care admission. Although it is primarily a clinical diagnosis, characterised by fluctuating weakness of ocular, bulbar, and limb muscles, confirmation is usually obtained through antibody testing and electrophysiological studies [4]. In the acute setting, a wide range of neurological symptoms, including those of MG, are frequently misattributed to stroke [6].

Bilateral ptosis has a broad differential, including mechanical, aponeurotic, myogenic, neuromuscular junction-related, neurogenic, and central causes. It is relatively uncommon, accounting for only 4% of cases, and may result from lesions in the frontoparietal or mesencephalic regions involving the oculomotor nerves. Although midbrain stroke is rare, representing only 1% of all strokes, paramedian midbrain lesions affecting the third nerve nucleus can produce bilateral ptosis [7].

This patient initially presented with bilateral ptosis. As his symptoms had been present in the community and resolved by the time of the emergency department visit on the same day, a working diagnosis of transient ischaemic attack was made. However, with further specialist input and careful review of the clinical history, including the precise timeline of symptoms, a diagnosis of MG was established. The patient’s steady improvement with intravenous IVIG and corticosteroid therapy supported this diagnosis, which was subsequently confirmed by the presence of acetylcholine receptor antibodies and a characteristic decremental response on electromyography.

A limitation of this case is that the initial emergency department evaluation did not include a detailed neurological examination, such as testing for fatigability of the ocular muscles, and collateral history was not explored to clarify symptom onset. Both may have supported an earlier diagnosis and highlight the importance of thorough clinical assessment in patients with fluctuating neurological symptoms.

Myasthenic crisis can occur spontaneously but is more commonly precipitated by identifiable triggers such as intercurrent infections, certain medications (e.g., aminoglycosides, fluoroquinolones, macrolides), or the initiation of high-dose corticosteroids for the treatment of MG [8]. In this case, there was no history of recent exposure to the aforementioned medications. In retrospect, the patient’s chest infection was the most likely precipitating factor, supported by the initial respiratory symptoms and chest X-ray evidence of consolidation.

## Conclusions

This case highlights how ocular symptoms of myasthenia gravis can mimic a neurovascular event, leading to initial misdiagnosis. Although bilateral ptosis is uncommon and should raise consideration of both neuromuscular and central causes, a thorough neurological examination, including assessment for fatigability, together with careful history-taking and collateral information, can help narrow the diagnosis at an earlier stage. Early involvement of the multidisciplinary team is also important in guiding investigations and management. Intercurrent infection remains a common precipitant of myasthenic crisis, and early immunomodulatory therapy with supportive care can result in substantial recovery.
